# General Self-Efficacy Mediates the Effect of Family Socioeconomic Status on Critical Thinking in Chinese Medical Students

**DOI:** 10.3389/fpsyg.2018.02578

**Published:** 2019-01-30

**Authors:** Lei Huang, Yun-Lin Liang, Jiao-Jiao Hou, Jessica Thai, Yu-Jia Huang, Jia-Xuan Li, Ying Zeng, Xu-Dong Zhao

**Affiliations:** ^1^Medical Education Division, Department of Psychiatry, Tongji Hospital, Tongji University School of Medicine, Shanghai, China; ^2^School of Medicine, Tongji University, Shanghai, China; ^3^University of Nebraska Medical Center, Omaha, NE, United States; ^4^Division of Medical Humanities and Behavioral Sciences, Tongji University School of Medicine, Shanghai, China; ^5^Shanghai East Hospital Affiliated to Tongji University, Shanghai, China; ^6^Pudong New Area Mental Health Center, Shanghai, China

**Keywords:** general self-efficacy, family socioeconomic status, critical thinking, medical students, personal traits

## Abstract

**Background:** Critical thinking (CT) is an essential competence for medical students. Family socioeconomic status (family SES) and general self-efficacy (GSE) play crucial roles in the development of CT. However, the association among family SES, GSE, and CT in Chinese medical students has yet to be fully investigated.

**Objectives:** To investigate the role of family SES and GSE in the development of CT in Chinese medical students.

**Methods:** 1,338 medical students were recruited using multistage stratified cluster sampling from three institutions in China. The Chinese critical thinking disposition inventory (CTDI-CV), General Self-Efficacy Scale (GSES), and a self-made inventory assessing family SES were administered to collect data. The relationship between CT and family SES as well as GSE was evaluated by structural equation modeling.

**Results:** Students of higher family SES obtained higher CTDI-CV and GSES scores. A positive correlation was found between family SES and CT (*r* = 0.101–0.141, *p* < 0.05 or *p* < 0.01), as well as between family SES and GSE (*r* = 0.111–0.129, *p* < 0.01). Moreover, GSE was moderately correlated with CT (*r* = 0.418, *p* < 0.01). The model of partial mediate effect of GSE showed the best fit index with *X*^2^ = 29.698, *df* = 9 and *X^2^/df* = 3.300, NFI = 0.990, IFI = 0.993, TLI = 0.984, CFI = 0.993, RMSEA = 0.041.

**Conclusion:** Family SES has a positive albeit limited influence on GSE and CT in Chinese medical students. GSE mediates the effect of family SES on CT and plays a larger role. Enhancing medical student’ GSE maybe an efficacious way to improve medical students’ CT.

## Introduction

Critical thinking (CT) is one of seven essential requirements for medical students (Core Committee Institute for International Medical Education, 2002). CT helps medical students argue independently, engage in judgment purposefully, and arrive at a well-reasoned resolution to a complicated problem (Allegretti and Frederick, 1995). CT has also been shown to positively correlate with academic performance in health professional students (Park and Kim, 2009; Chang et al., 2011; Pardamean, 2012). In higher medical education, several factors have been identified to influence medical students’ CT including education and experience (Terenzini et al., 1995; Pascarella et al., 1996; Tian and Low, 2011), teaching methods (Popil, 2011; Soucy, 2011; Huang et al., 2012; Wahl and Thompson, 2013), and learning style (Myers and Dyer, 2006; Andreou et al., 2014). Apart from educational factors, demographic and sociologic factors (i.e., age, gender, race, religion and marriage) are also associated with CT in medical students. In addition, previous studies have shown that personal characteristics such as cognition (Magno, 2010; Başbay, 2013), self-efficiency (Dehghani et al., 2011b; Gloudemans et al., 2013), personality traits (Clifford et al., 2004; Friedman, 2004; Ku and Ho, 2010), and environmental factors such as culture (Grosser and Lombard, 2008; Lun et al., 2010), family background (Chau-Klu et al., 2001), and work atmosphere (Mcallister and Mckinnon, 2009), are significantly associated with the development of CT. The relationships between CT and influential factors such as educational, demographic, sociologic, and personal characteristics have been discussed extensively. However, there are very few studies on the association of medical students’ CT with family related factors.

Family socioeconomic status is one indicator of family resources that has a significant impact on the domestic atmosphere, family members’ interpersonal relationships, and parental rearing styles. As a result, family SES may impact children’s development both directly and indirectly. Family SES is not a single, individual trait, but an integration of multiple social circumstances that a child/young adult may be exposed to. Family income, parental education, and occupation are generally used to represent the multidimensional variable family SES (White, 1982; Matthews and Gallo, 2010). Hill suggested that parental SES has a direct influence on a child’s eventual occupational attainment and is the most powerful and consistent predictor of career aspiration and achievement (Hill et al., 2004). Fan also proposed the importance of a mother’s family SES on medical students and its influence on stress, mental disturbances, attitude toward life, personality, health, discipline, internationalization, and professionalism (Fan et al., 2007). One study on university students also found that students of bourgeois or upper-class families or fathers excelled in CT compared to students of lower classes and that the availability of family resources may explain the effects of class on students’ CT predisposition. Furthermore, the association between family SES and health problems (i.e., growth retardation, diseases, injuries, cognitive and academic attainment, and socio-emotional development) may already be unwinding prior to birth and extend through adulthood (Bradley and Corwyn, 2002). Research also demonstrates a positive relationship between low family SES and low scholastic achievement/IQ in childhood (Pianta et al., 1990; Alexander et al., 1993; Duncan et al., 2010).

General self-efficacy (GSE) is one potential personal factor which may influence CT in medical students. GSE is refined when one competes in life for success across a wide range of domains and tasks. Studies have shown that GSE plays a potent role in judgment, thinking, problem solving (Pajares, 1997; Dweck and Leggett, 1999) and academic success (Pajares and Valiante, 1997; Pajares et al., 1999). Self-efficacy beliefs as a motivational construct has a critical role in the development of CT (Artino and Stephens, 2009). A positive correlation between GSE and medical students’ CT has been shown in studies, which recommended considering self-efficacy as a motivational factor for developing learners’ CT skills (Dehghani et al., 2011b; Shanghai and Zhang, 2011; Huang et al., 2015a). Furthermore, evidence derived from SEM and analytical procedures highlighted that GSE helped mediate both goals and CT (Phan, 2009) and that GSE may predict other factors affecting CT. Four of these factors which may affect GSE include attainment, experience, social persuasion, and physiological factors (Bandura, 1995). Interestingly, these four factors may all be affected by family SES as well. Studies have also shown that low family SES college students scored significantly lower than their peers on GSE (Tong and Song, 2004). Additionally, mother’s education level and family’s current affluence were independent predictors of GSE for adolescents (Mazur et al., 2014).

Previous research found limited effects of educational environment on students’ CT improvement. Instead, it has been suggested that family class background has a significant influence on university students’ CT (Inman and Pascarella, 1998). Moreover, a large number of studies have suggested the potential impact of family SES on children’s CT development, demonstrating a positive association between family SES and GSE as well as GSE and CT for medical students. Through deductive reasoning, we hypothesize that GSE may mediate the effects of family SES on CT in medical students. Previous research has been based on university students with a focus on family resources with little research on how family SES influences medical students’ CT via personal development. This present study adds a unique perspective to research on medical students’ CT. The purpose of this study was (1) to assess links between family SES and GES as well as between GES and CT for medical students; (2) to explore the mediating role of GES on family SES and CT. Research on family SES is of high educational concern but is rarely taken into consideration. The findings of this study may help medical educators better understand medical students from diverse family SES origins, allowing educators to provide a greater commitment to CT and to effectively cultivate medical students’ CT.

## Materials and Methods

### Participants

1,338 Chinese medical students were enrolled in this study. Students were sampled using multistage stratified cluster sampling from three medical schools, Tongji University School of Medicine (Shanghai), Medical College of Soochow University (Jiangsu Province), and Gannan Medical University (Jiangxi Province) in China. Among these students, 667 were male (49.9%) and 671 were female (50.1%). Two hundred and ninety five were first-year students (22.0%), 252 were second-year students (18.8%), 300 were third-year students (22.4%), 289 were fourth-year students (21.6%), and 202 were interns (15.1%). Ages ranged from 15 to 27 years old (22.8 ± 1.74 years).

### Procedure

Firstly, a questionnaire was organized via two standardized assessment tools and self-made questions which included family SES and sociodemographic information. Secondly, prior to the formal study, 20 medical students at Tongji University School of Medicine were invited to complete this questionnaire as a pilot survey. Then, the questionnaire was modified according to the feedback from students to ensure that all the questions were understandable and unambiguous. Thirdly, the modified version of questionnaire and consent form were printed and delivered to participants by three staff from institutions’ medical education offices. To ensure participant confidentiality, we provided a return envelope for participants to seal finished questionnaires. Participants were asked to complete and return the survey in an upcoming meeting 2 weeks later. We encouraged their involvement and reminded them to return the finished questionnaire 3 days before the deadline. Upon all of the questionnaires completion and return, two members of our team inputted data into Excel and transformed it to SPSS. Data analysis was guided by the statistician in our institution. The project was approved by the Ethics Review Committee of Tongji Hospital and Tongji University (Registration Number K-2014-020).

### Assessment Tools

#### Chinese Critical Thinking Disposition Inventory (CTDI-CV)

The CTDI-CV inventory was translated and modified based on the California Critical Thinking Disposition Inventory (TDI) (Facione and Facione, 1992) by Peng (Yeh, 2002). The inventory measures CT in Chinese medical students via 70 items aimed at measuring the seven dimensions of CT: truth seeking, analyticity, open-mindedness, systematicity, inquisitiveness, self-confidence, and cognitive maturity. The overall content validity index (CVI) is 0.89, with the subscale CVI ranging from 0.6 to 1. The overall alpha is 0.90, and the subscale alpha ranges from 0.54 to 0.77. Higher scores on each dimension or a higher total score reflects better CT ability.

#### Family SES Survey

This survey consisted of three sections intended to record medical students’ personal data including family-economic-condition, parental education, and parental occupation. In the family-economic-condition section, students were asked to subjectively evaluate their family income as “low,” “average,” or “affluent.” The other two sections recorded data regarding parental education classified as “below high school” or “high school or above” and parental occupation classified as “non-high-tech (labor, farmer, migrant worker, ordinary staff, and service provider, driver)” or “high-tech (teacher, doctor, senior professional and technical personnel, corporate manager)” (Shi and Shen, 2007).

#### General Self-Efficacy Scale (GSES)

This scale was developed by Schwarzer and Born (1997). It has been translated into 30 languages and is widely implemented worldwide (Schwarzer et al., 1997). A Chinese version, adapted by Wang, was used in this study and consisted of 10 questions. The scale’s Cronbach’s Alpha is 0.87, split-half reliability coefficient is 0.82, and test-retest reliability coefficient is 0.83 (Wang et al., 2001).

#### Sociodemographic Data Collection Form

This form was used to collect sociodemographic data from medical students participants including gender, age, grade, and college affiliation.

### Statistical Analyses

Statistical analyses were performed using SPSS software version 19.0 (SPSS Inc., Chicago, IL, United States). Results are displayed as mean, standard deviation (SD), and percentage. Data were compared and analyzed using either the two tailed Student’s *t*-test, One-way ANOVA test, or Pearson’s correlation-test, unless otherwise stated. AMOS20.0 was used to establish SEM and was applied to analyze the mediating role of GSE between family SES and CT. Statistical significance was noted at *p* < 0.05, *p* < 0.01, and *p* < 0.001.

## Results

### Correlations Between Family SES and CT as Well as Between Family SES and GSE

Chinese critical thinking disposition inventory scores ranged from 205 to 383 (287.20 ± 29.67). GSES scores ranged from 10 to 40 (25.91 ± 4.75). As shown in Supplementary Table 1, students who scored higher on the CTDI-CV and GSES were significantly more likely to have a parent with a “high school or above” education and a “high-tech” occupation. GSES scores among students from “affluent,” “average,” and “low” income families also significantly differed. Interestingly, students from “average” income families rather than “low” income families had the lowest scores. No significant differences in CTDI-CV score were found between students from “low,” “average,” and “affluent” families, and between males and females in this study.

Supplementary Table 2 showed the correlation coefficients between family SES variables and GSES and CTDI-CV scores. Significant positive correlations were found among all family SES variables and GSES score. Significant correlations were also found between parental education and CTDI-CV score as well as parental occupation and CTDI-CV score. However, no significant correlation was found between family-economy-condition and CTDI-CV score. GSES score was also significantly positively correlated with CTDI-CV score.

### GSE’s Mediating Effect Between Family SES and CT

**FIGURE 1:**
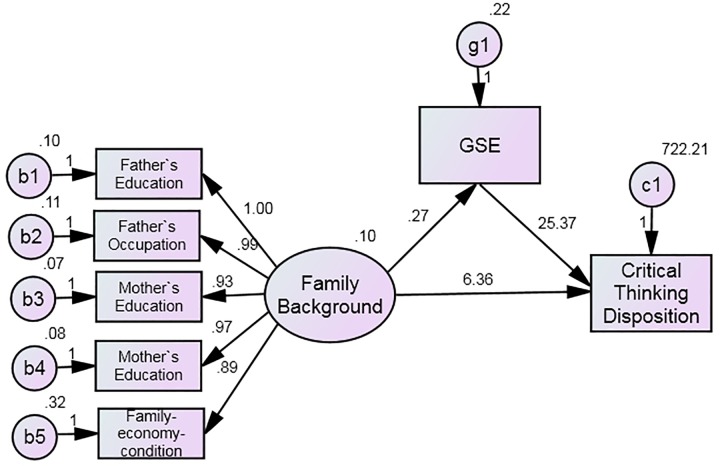
Hypothesized SEM of the possible relationships among family SES, GSE, and CT.

Using SEM, three models were established to elucidate possible relationships among family SES, GSE, and CT (Figure 1). In Model 1, family SES exerted its impact on CT in two ways: directly (family SES without any mediation) and indirectly (family SES with GSE mediation). In Model 2, family SES exerted an indirect effect on CT (with GSE mediation). In Model 3, family SES exerted a direct effect on CT (without any mediation).

The model fit indices and comparisons are listed in Supplementary Table 3. *X^2^*, *X^2^/df*, NFI, IFI, TLI, CFI, RMSEA are common SEM fitting optimization indices. Because the SEM statistic *X^2^* is highly sensitive to sample size, significance is more easily obtained when *n* > 200. The sample size in this study is 1,338, the SEM statistic *X^2^/df* is best able to determine model fit. *X^2^/df* < 5 is generally considered a good fit.

Compared with Model 3, Model 1 had a higher *X^2^* value but lower *X^2^/df* and *RMSEA* values. Model 2’s *X^2^*, *X^2^/df*, and *RMSEA* values were higher than Model 1. The additional indices NFI, IFI, TLI, and CFI were not significantly different between the three models. As a result, SEM data favored Model 1 with *X*^2^= 29.698, *df* = 9, *X^2^/df* = 3.300, NFI = 0.990, IFI = 0.993, TLI = 0.984, CFI = 0.993, RMSEA = 0.041, and rejected both Model 2 and Model 3.

Model 1 was then further investigated. Supplementary Table 4 shows the path coefficients relating the three factors in Model 1. Family SES was a positive contributor to GSE (β = 0.179, *p* < 0.001) and CT (β = 0.067, *p* < 0.01). GSE was also a positive contributor to CT (β = 0.406, *p* < 0.001).

Supplementary Table 5 shows the path coefficients of each individual Family SES variable in Model 1. Weighting of all subscales were statistically significant (*p* < 0.001) with regards to all five formative-indicator constructs influencing family SES. From the highest to lowest weighting was mother’s occupation (β = 0.734, *p* < 0.001), mother’s education (β = 0.729, *p* < 0.001), father’s education (β = 0.700, *p* < 0.001), father’s occupation (β = 0.684, *p* < 0.001) and family-economic-condition (β = 0.443, *p* < 0.001).

## Discussion

There is a lack of research examining the relationship between family SES and GSE as well as CT in medical students. In this study, we found a positive, although weak correlation, between family SES and GSE as well as between family SES and CT in Chinese medical students (all Pearson correlation r values were under 0.3). Among study participants, those from high SES families had higher GSE and CT scores. These results echo a study performed in mainland China, which showed that family SES has a positive effect on GSE and found that college students from lower SES families had lower GSE scores and lower subjective well-being (Tong and Song, 2004). Similarly, another study in Hong Kong suggested that compared to those from of lower class families, students from upper class families exhibited higher CT skills (Chau-Klu et al., 2001). The family environment is the first and one of the most important learning settings to which children are exposed. As such, family SES has potential effects on an individuals’ physical and mental development and may exert a influence on GSE and CT in the long term. Previous studies have suggested that low family SES increased the risk of stress/adversity exposures, including traumatic life events, chronic stress, perceived stress, and daily hassles (Gallo et al., 2005; Hatch and Dohrenwend, 2007), which fosters negative emotions and psychological distress, such as anxiety, depressive symptoms, psychological disorders, hostile cognition, and anger (Gallo and Matthews, 2003). Additionally, individuals from lower SES families generally have fewer tangible/interpersonal resources and intrapersonal relationships to cope with stressful events when they are exposed to situations in which the utilization of resources is obligatorily required. In contrast, students from higher SES families enjoy better educational resources and opportunities, whereas lower SES students often must face economic or other family pressures which may give rise to a negative attitude (Randolph et al., 2006; Matthews and Gallo, 2010). These findings suggest that family SES influences a young person’s living environment and learning experiences, which subsequently affect GSE and CT and consequently, academic performance.

Family SES, as one indicator of family resources, has a meaningful impact on family atmosphere, family members’ relationships, and parental rearing style. It may also indirectly impact the development of a child’s self-efficacy. Previous studies have revealed that relationships among family members may influence GSE in young adulthood (Brown, 1998). Furthermore, parental rearing style is a distinct predictor of CT in medical students (Huang et al., 2015b). This may be why the path coefficients as determined by SEM analysis showed a limited effect of family SES on GSE (β = 0.179) and CT (β = 0.067). We assumed that other independent factors including family atmosphere, parent-child relationships, parental rearing style, family members, and family social context may also play a role beyond the framework of the family and contribute to children’s GSE and CT.

Model 1 was determined to be the best fit by SEM analysis, which was consistent with our initial hypothesis that GSE mediates the effect of family SES on CT in Chinese medical students. This finding is similar to previous studies, which revealed that maternal education and a family’s level of wealth were both independent predictors of adolescents’ GSE and that self-efficacy was a mediator between SES and life quality (Mazur et al., 2014). Furthermore, our study also revealed that GSE exerted a positive effect on CT in medical students. This finding echoed previous research conducted by Marzieh Dehghani, which suggested that GSE is a motivational factor during the development of CT among university students (Dehghani et al., 2011a). Similar conclusions were found in two additional studies in which the subjects were Chinese medical students and nurses (Zhang et al., 2001; Baldwin, 2015).

GSE is positively related to university students’ creative personalities, especially in the aspects of adventure, challenge and curiosity (Myers, 1992). Therefore, GSE plays a central role in the development of CT skills. People with higher self-efficacy levels often possess enhanced sense of self-control. They prefer to choose challenging tasks and goals, persist in their academic performance, and confront difficult situations instead of avoiding them (Bandura and Locke, 2003; Mclaughlin et al., 2010). Additionally, students with higher levels of self-efficacy can better apply higher level learning strategies to their studies (Shu-Ling and Pei-Yi, 2008). This further improves their academic performance, thinking style, logic and analyticity, and courage to seek truth. These improvements benefit their CT ability in the long-run as good academic performance provides positive feedback to further enhance self-efficacy.

In this study, we demonstrated that family SES had a positive correlation with GSE and CT, although the contribution is limited. GSE mediated the effect of family SES on CT and had a stronger contribution to medical students’ CT compared to family SES. These results supported our hypothesis that family related factors would influence medical students’ CT via personal factors. However, although our findings agreed with the past study on Hong Kong university students, our findings on the relationship between family SES with both GSE and CT are less pronounced than this previous study which strongly emphasized the role of family class and resources. Implications of this study suggest that students from lower family SES may still score as well as students from higher family SES in CT performance if more attention is directed toward improving medical students’ GSE and consequently CT. Nonetheless, other personal factors, such as cognition and personality traits, may also mediate the effect of other family related factors and play an important role in medicals students’ CT. These variables need to be explored further to help improve knowledge on CT to improve medical student education and medical students’ future careers as physicians.

### Strengths and Limitations

To our knowledge, this is the first study to explore the mediating role of GSE between family SES and CT in Chinese medical students, which helps us better understand and improve medical students’ CT. Furthermore, the sample of participants was sizable. However, there are still limitations to this study. Firstly, the sample is from three medical schools in China and may not be adequately representative of its student population in the absence of strictly random sampling. Secondly, all data are obtained from self-assessment. Therefore, inaccuracies caused by memory biases and subjective attitudes were unavoidable. Thirdly, family SES data was obtained by self-assessment without national objective standards, due to the vastly different levels of economic development across the different regions of China. Moreover, other family factors which could influence the development of CT, such as number of siblings, social context, presence of grandparents, uncles, or other extended family members in the family environment have not been mentioned in this study. Finally, this study is cross-sectional and descriptive in nature, while family SES and self-efficacy are constantly changing across one’s life. Therefore, a longitudinal and prospective study may be needed for further evaluation.

## Conclusion

In conclusion, family SES has a positive but limited influence on GSE and CT in Chinese medical students. GSE mediates the effect of family SES on CT and plays a larger role than family SES on medical students’ CT. Consequently, improving medical students’ GSE may be an efficacious method to improve medical students’ CT.

## Ethics Statement

This study was approved by the Ethics Review Committee of Tongji Hospital and Tongji University (Registration Number K-2014-020).

## Author Contributions

LH contributed to study design, recruitment of participants, data analysis, and interpretation and writing of the manuscript. Y-LL assisted in the data acquisition and interpretation of results. J-JH and JT assisted in the interpretation of the results and revision of the manuscript. Y-JH assisted in the data acquisition and concept of the design. J-XL assisted in the data analysis. YZ assisted in recruitment of participants. X-DZ contributed to study design and supervision. All authors have approved the final manuscript.

## Conflict of Interest Statement

The authors declare that the research was conducted in the absence of any commercial or financial relationships that could be construed as a potential conflict of interest.

## Abbreviations

CT: critical thinking
CTDI-CV: Chinese critical thinking disposition inventory
CFI: comparative fit index
family SES: family socioeconomic status
GSE: general self-efficacy
GSES: General Self-Efficacy Scale
IFI: incremental fix index
NFI: Bentler-Bonett normed fit index
RMSEA: root mean square error of approximation
SEM: structural equation modeling
TLI: Tucker-Lewis index
