# Supplementary material for: General Self-Efficacy Mediates the Effect of Family Socioeconomic Status on Critical Thinking in Chinese Medical Students
Source: Front Psychol. 2019 Jan 30;9:2578. doi: 10.3389/fpsyg.2018.02578 (PMC6363706; doi:10.3389/fpsyg.2018.02578)
Supplement: Supplementary file 1 [file Table_1.DOC]

Supplementary Material

# General Self-Efficacy Mediates the Effect of Family Socioeconomic Status on Critical Thinking in Chinese Medical Students

**Lei Huang^1,2^, Yun-Lin Liang^2^, Jiao-Jiao Hou^2^, Jessica Thai^3^, Yu-Jia Huang^2^, Jia-Xuan Li^2^,Ying Zeng^2^,Xu-DongZhao^4,5,6*^**

**Correspondence:** Prof. Xu-Dong Zhao E-mail: zhaoxd62@gmail.com

**Table1 Comparison of CTDI-CV and GSES score among different family SES variables**

| Variables | n | % | CTDI-CV (Mean ± SD) | Test *p-value* | GSES (Mean ± SD) | Test *p-value* |
| --- | --- | --- | --- | --- | --- | --- |
| Family-economic-condition | |  |  |  |  |  |
| Low | 265 | 19.8 | 288.54 ± 30.44 | *F=1.963* | 25.69 ± 4.54 | *F=16.28* |
| Average | 813 | 60.8 | 285.94 ± 28.57 | *p=0.141* | 25.50 ± 4.66 | *p=0.000* |
| Affluence | 260 | 19.4 | 289.75 ± 32.06 |  | 27.39 ± 4.97 |  |
| Father's education |  |  |  |  |  |  |
| Below High school | 969 | 72.4 | 284.62 ± 28.31 | t=-5.194 | 25.55 ± 4.51 | t=-4.434 |
| High school or above | 369 | 27.6 | 293.96 ± 32.05 | *p*=0.000 | 26.83 ± 5.21 | *p*=0.000 |
| Father's occupation |  |  |  |  |  |  |
| Non high-tec | 951 | 71.1 | 284.53 ± 28.55 | t=-5.209 | 25.52 ± 4.61 | t=-4.755 |
| High-tec | 387 | 28.9 | 293.76 ± 31.35 | *p*=0.000 | 26.87 ± 4.94 | *p* = 0.000 |
| Mother's education |  |  |  |  |  |  |
| Below High school | 1071 | 80 | 285.70 ± 29.06 | t=-3.317 | 25.62 ± 4.57 | t=-4.431 |
| High school or above | 267 | 20 | 293.21 ± 31.36 | *p* =0.000 | 27.05 ± 5.26 | *p*=0.000 |
| Mother's occupation |  |  |  |  |  |  |
| Non high-tech | 1047 | 78.3 | 286.21 ± 29.16 | t=-2.310 | 25.60 ± 4.56 | t=.-4.550 |
| High-tech | 291 | 21.7 | 290.75 ± 31.26 | *p*=0.000 | 27.02 ± 5.23 | *p*=0.000 |
| Abbreviations: n, number; *t* , Student’s *t*-test ;F, One-way ANOVA test. | | | | | |  |
