# Supplementary material for: General Self-Efficacy Mediates the Effect of Family Socioeconomic Status on Critical Thinking in Chinese Medical Students
Source: Front Psychol. 2019 Jan 30;9:2578. doi: 10.3389/fpsyg.2018.02578 (PMC6363706; doi:10.3389/fpsyg.2018.02578)
Supplement: Supplementary file 2 [file Table_2.DOCX]

Supplementary Material

General Self-Efficacy Mediates the Effect of Family Socioeconomic Status on Critical Thinking in Chinese Medical Students

**Lei Huang^1,2^, Yun-Lin Liang^2^, Jiao-Jiao Hou^2^, Jessica Thai^3^, Yu-Jia Huang^2^, Jia-Xuan Li^2^,Ying Zeng^2^,Xu-DongZhao^4,5,6*^**

**Correspondence:** Prof. Xu-Dong Zhao E-mail: zhaoxd62@gmail.com

| **Table2 Analytical data of correlation coefficient** | | | | | | | |
| --- | --- | --- | --- | --- | --- | --- | --- |
| Variables | Family-economic condition | Father's education | Father's occupation | Mother's education | Mother's occupation | CT | GSE |
|  |  |  |  |  |  |  |  |
| CT | 0.012 | 0.141^**^ | 0.141^**^ | 0.101^**^ | 0.063^*^ | 1 |  |
| GSE | 0.111^**^ | 0.120^**^ | 0.129^**^ | 0.120^**^ | 0.124^**^ | 0.418^**^ | 1 |
| * *p*<0.05;** *p*<0.01 | | | | |  |  |  |
