# Supplementary material for: General Self-Efficacy Mediates the Effect of Family Socioeconomic Status on Critical Thinking in Chinese Medical Students
Source: Front Psychol. 2019 Jan 30;9:2578. doi: 10.3389/fpsyg.2018.02578 (PMC6363706; doi:10.3389/fpsyg.2018.02578)
Supplement: Supplementary file 3 [file Table_3.DOCX]

Supplementary Material

General Self-Efficacy Mediates the Effect of Family Socioeconomic Status on Critical Thinking in Chinese Medical Students

**Lei Huang^1,2^, Yun-Lin Liang^2^, Jiao-Jiao Hou^2^, Jessica Thai^3^, Yu-Jia Huang^2^, Jia-Xuan Li^2^,Ying Zeng^2^,Xu-DongZhao^4,5,6*^**

**Correspondence:** Prof. Xu-Dong Zhao E-mail: zhaoxd62@gmail.com

| **Table 3 Index of Model fit and models’ comparison** | | | | | | | | | |
| --- | --- | --- | --- | --- | --- | --- | --- | --- | --- |
| Model | *χ^2^* | *df* | *χ^2^/df* | NFI | IFI | TLI | CFI | RMSEA | |
| Model1:partial mediate effect | 29.698 | 9 | 3.3 | 0.99 | 0.993 | 0.984 | 0.993 | | 0.041 |
| Model2:full mediate effect | 34.76 | 10 | 3.476 | 0.988 | 0.992 | 0.983 | 0.992 | | 0.043 |
| Model3:no mediate effect | 21.84 | 5 | 4.368 | 0.992 | 0.994 | 0.981 | 0.994 | | 0.050 |
