# Supplementary material for: General Self-Efficacy Mediates the Effect of Family Socioeconomic Status on Critical Thinking in Chinese Medical Students
Source: Front Psychol. 2019 Jan 30;9:2578. doi: 10.3389/fpsyg.2018.02578 (PMC6363706; doi:10.3389/fpsyg.2018.02578)
Supplement: Supplementary file 4 [file Table_4.DOCX]

Supplementary Material

General Self-Efficacy Mediates the Effect of Family Socioeconomic Status on Critical Thinking in Chinese Medical Students

**Lei Huang^1,2^, Yun-Lin Liang^2^, Jiao-Jiao Hou^2^, Jessica Thai^3^, Yu-Jia Huang^2^, Jia-Xuan Li^2^,Ying Zeng^2^,Xu-DongZhao^4,5,6*^**

**Correspondence:** Prof. Xu-Dong Zhao E-mail: zhaoxd62@gmail.com

Table 4 Path coefficient among three factors in Model 1

| Structural paths | *b* | *β* | *S.E.* | *C.R.* |
| --- | --- | --- | --- | --- |
| GSE Family SES | 0.272 | 0.179 | 0.049 | 5.561^***^ |
| CT Family SES | 6.360 | 0.067 | 2.785 | 2.284^**^ |
| CT GSE | 25.366 | 0.406 | 1.583 | 16.019^***^ |

*b*, unstandardized coefficients; β, standardized coefficients; S.E, standard error; C.R., critical ratio; **, *p*<0.01; ***, *p*<0.001.
