# Supplementary material for: General Self-Efficacy Mediates the Effect of Family Socioeconomic Status on Critical Thinking in Chinese Medical Students
Source: Front Psychol. 2019 Jan 30;9:2578. doi: 10.3389/fpsyg.2018.02578 (PMC6363706; doi:10.3389/fpsyg.2018.02578)
Supplement: Supplementary file 5 [file Table_5.DOCX]

Supplementary Material

General Self-Efficacy Mediates the Effect of Family Socioeconomic Status on Critical Thinking in Chinese Medical Students

**Lei Huang^1,2^, Yun-Lin Liang^2^, Jiao-Jiao Hou^2^, Jessica Thai^3^, Yu-Jia Huang^2^, Jia-Xuan Li^2^,Ying Zeng^2^,Xu-DongZhao^4,5,6*^**

**Correspondence:** Prof. Xu-Dong Zhao E-mail: zhaoxd62@gmail.com

**Table 5 Path coefficients within all family SES variables in Model 1**

| Structural paths | *b* | *Β* | *S.E.* | *C.R.* |
| --- | --- | --- | --- | --- |
| Family economic conditions Family SES | 0.887 | 0.443 | 0.071 | 12.521^***^ |
| Father's education Family SES | 1.000 | 0.700 |  |  |
| Father's occupation Family SES | 0.990 | 0.684 | 0.061 | 16.203^***^ |
| Mother's education Family SES | 0.931 | 0.729 | 0.058 | 15.955^***^ |
| Mother's occupation Family SES | 0.968 | 0.734 | 0.078 | 12.468^***^ |

b, unstandardized coefficients; β, standardized coefficients; S.E, standard error; C.R., critical ratio; **, *p*<0.01; ***, *p*<0.001.
